# Flagellar Movement in Two Bacteria of the Family *Rickettsiaceae*: A Re-Evaluation of Motility in an Evolutionary Perspective

**DOI:** 10.1371/journal.pone.0087718

**Published:** 2014-02-05

**Authors:** Claudia Vannini, Vittorio Boscaro, Filippo Ferrantini, Konstantin A. Benken, Timofei I. Mironov, Michael Schweikert, Hans-Dieter Görtz, Sergei I. Fokin, Elena V. Sabaneyeva, Giulio Petroni

**Affiliations:** 1 Biology Department, University of Pisa, Pisa, Italy; 2 Department of Cytology and Histology, St.-Petersburg State University, St.-Petersburg, Russia; 3 Biological Institute, Stuttgart University, Stuttgart, Germany; 4 Department of Invertebrate Zoology, St.-Petersburg State University, St.-Petersburg, Russia; University of North Dakota School of Medicine and Health Sciences, United States of America

## Abstract

Bacteria of the family *Rickettsiaceae* have always been largely studied not only for their importance in the medical field, but also as model systems in evolutionary biology. In fact, they share a recent common ancestor with mitochondria. The most studied species, belonging to genera *Rickettsia* and *Orientia*, are hosted by terrestrial arthropods and include many human pathogens. Nevertheless, recent findings show that a large part of *Rickettsiaceae* biodiversity actually resides outside the group of well-known pathogenic bacteria. Collecting data on these recently described non-conventional members of the family is crucial in order to gain information on ancestral features of the whole group. Although bacteria of the family *Rickettsiaceae*, and of the whole order *Rickettsiales*, are formally described as non-flagellated prokaryotes, some recent findings renewed the debate about this feature. In this paper we report the first finding of members of the family displaying numerous flagella and active movement inside their host cells. These two new taxa are hosted in aquatic environments by protist ciliates and are described here by means of ultrastructural and molecular characterization. Data here reported suggest that the ancestor of *Rickettsiales* displayed flagellar movement and re-evaluate the hypothesis that motility played a key-role in the origin of mitochondria. Moreover, our study highlights that the aquatic environment represents a well exploited habitat for bacteria of the family *Rickettsiaceae*. Our results encourage a deep re-consideration of ecological and morphological traits of the family and of the whole order.

## Introduction

The family *Rickettsiaceae* (Pinkerton, 1936 [1], emended by Dumler and colleagues [2]) represents one of the most studied taxonomic groups of bacteria. At present it includes members of the genera *Rickettsia* and *Orientia*, and the recently described candidate genera “*Candidatus* Cryptoprodotis” and “Candidatus Megaira” [3], [4]. Bacteria belonging to this family have attracted a great deal of attention and have been traditionally studied with a medical-entomological approach. The reason for such a high interest in these microorganisms resides mainly in the fact that species of the genera *Rickettsia* and *Orientia* are well-known etiological agents of many human and vertebrate diseases, having blood-feeding arthropods as vectors. Although most studies were mainly focused on pathogenic members of the family, an increasing number of non-pathogenic bacteria have been described also in non-hematophagous arthropods [5]–[9], and even associated with completely different organisms like leeches [10], cnidarians [11], [12], green algae [13] and protozoa [3], [4], [14]–[16]. Thus, it is now obvious that a large fraction of *Rickettsiaceae* biodiversity resides outside the group of pathogenic bacteria hosted by blood-feeding arthropods. It is then noteworthy that, among protists, ciliates harbor not only bacteria of the family *Rickettsiaceae* [3], [4], [15], but also several bacteria belonging to other groups of the order *Rickettsiales* [17]–[23]. Thus, these eukaryotic microorganisms can reasonably be considered useful biological systems for investigations on rickettsial biodiversity.

Besides their interest for the medical field, bacteria of the family *Rickettsiaceae* and of the whole order *Rickettsiales* assume additional importance. In the context of evolutionary biology, the study of these bacteria represents a key aspect in order to gain a better knowledge on one of the most fundamental steps in the history of life on earth: the evolution of mitochondria. It is now widely accepted that mitochondria originated from endosymbiotic prokaryotes [24], and that *Rickettsiales* bacteria and mitochondria share a common ancestor. Although the debate is still open [25], [26], this hypothesis is strongly supported by molecular data [27]–[31] and by other features of *Rickettsiales* such as the endosymbiotic lifestyle, the presence of similar respiratory chains and the lack of alternative energy patterns (for reviews see [32], [33]). *Rickettsiales* bacteria represent the closest extant relatives of the mitochondria ancestor. Therefore, the study of this group of prokaryotes can provide important indications concerning the origin of the organelle.

The majority of available data refers mainly to louse- or tick-borne rickettsial human pathogens [34]. As these species are highly specialized organisms co-evolved with their hosts, they are probably quite divergent from the rickettsial-mitochondrial common ancestor [35]. It is thus possible that some crucial evolutionary adaptations were overlooked because they are no longer retained in the deeply investigated extant species of *Rickettsiales.* According to some authors, protists could be considered as the ancestral hosts of members of the whole order *Rickettsiales* [33]. In this case, the later specialization to multicellular eukaryotic hosts coincided probably with the beginning of genome reduction in the symbionts [33]. Therefore, investigations on protist-borne *Rickettsiales* bacteria could provide new insights on ancestral features of the whole group.

A critical feature for understanding the evolutionary history of *Rickettsiales* and the steps which led to the establishment of the mitochondrial symbiosis is the presence or absence of movement in the mitochondria ancestor. Indeed, this feature could discriminate between a passive model of engulfment or a model implying a more active role of the bacterial partner [36], [37]. It has been shown that some species of *Rickettsia* can induce actin polymerization and move by generating actin filaments that resemble those present in filopodia [38]–[40]. The same capacity has been shown for members of the family *Holosporaceae* (*Rickettsiales*) [41]. Nevertheless, although the presence of some extracellular structures like pili or fimbriae-like elements has been demonstrated in the *Rickettsiaceae* [40], [42], [43], flagella have never been observed in members of the family. Bacteria of the order *Rickettsiales* were officially described as “bacteria with … no flagella” [44] and no evidence of active movement has been reported until now.

The recent finding of a complete set of flagellar genes in “*Candidatus* Midichloria mitochondrii”, a tick symbiont belonging to the order (family “*Candidatus* Midichloriaceae” [45]), strongly challenged the firm belief that *Rickettsiales* never displayed flagellar movement during their evolutionary history [46]. Indeed, genomic data obtained from “*Ca.* M. mitochondrii” show a pattern of vertical inheritance for the flagellar genes and strongly support the hypothesis that these genes were present in the ancestor of *Rickettsiales* and mitochondria as well. Although seven of these genes are expressed at the RNA level, the presence of flagella and/or of active movement has never been observed in “*Ca.* M. mitochondrii” [47]. Actual peritrichous flagella are present in the ciliate endosymbiont *Lyticum*, a close relative of “*Ca*. M. mitochondrii”, but this bacterium is nevertheless completely unable to move and probably maintained these structures for a different function [48].

In this paper we report the first finding of members of the family *Rickettsiaceae* displaying numerous flagella and active movement inside their host cells. These two new taxa of bacterial endosymbionts of protist ciliates are described by means of ultrastructural and molecular characterization.

## Materials and Methods

### Ciliate Host Isolation, Culturing and Identification

The study was performed on the ciliates listed in Table 1. Ciliate cultures were maintained in one of the following media: Synthetic Medium for Blepharisma (SMB), infusion of Cerophyll (Cerophyll Laboratories, Kansas City, Missouri, USA) and straw, lettuce infusion and artificial brackish water (5‰ salinity). They were periodically fed with bacteria (*Raoultella planticola* or *Enterobacter aerogenes*) or the photosynthetic flagellate *Dunaliella tertiolecta*.

**Table 1:** Main features of the two newly described bacterial species, as well as data of their hosts.

Taxon	Host	Origin	Strain orpopulation	Intracellularlocalization	Flagella	Viral capsid-likestructures	Insertions
**“** ***Candidatus*** ** Trichorickettsia** **mobilis”**	*Paramecium* *multimicronucleatum*	Italy, Serchio River(freshwater pond)	PS23(polyclonal)	Macronucleus	YES	YES,cylindrical	Long
	*Paramecium* *multimicronucleatum*	Italy, Lucca (canal)	LSA(monoclonal)	Macronucleus	YES	YES,cylindrical	Long
	*Paramecium* *multimicronucleatum*	Germany, Büsnau(wastewater plant)	Pm(monoclonal)	Macronucleus	YES	YES,cylindrical	Long
	*Paramecium* *nephridiatum*	Italy, Latina(wastewater plant)	PAR(polyclonal)	Cytoplasm	NO	YES,icosahedral	Long
	*Euplotes aediculatus*	India, New Delhi(freshwater pond)	In(polyclonal)	Cytoplasm	NO	NO	Long
**“** ***Candidatus*** **Gigarickettsia** **flagellata”**	*Spirostomum minus*	Italy, Serchio River(freshwater pond)	SS03(polyclonal)	Cytoplasm	YES	NO	Short

Identification of the ciliate species was achieved in previous studies by Boscaro and colleagues [23], [49] or in this study by characterization of 18S rRNA gene sequence (see below) and by morphological observations according to Fokin [50]. For the morphological determination, living observations of the ciliates as well as checking of fixed material, stained by Feulgen reaction, were performed.

### *In vivo* Observations and Transmission Electron Microscopy (TEM)

*In vivo* observations were performed using differential interference contrast (DIC) microscopy with a Leitz or Leica (Weitzlar, Germany) microscope at a magnification of 40–1250 X.

TEM preparations were obtained by fixing ciliate cells in glutaraldehyde and/or paraformaldehyde in cacodylate or phosphate buffer, with a postfixation in OsO_4_. The cells were then embedded in Epoxy embedding medium (Fluka, BioChemika) and cut using a Reichert-Jung Ultracut E or a LKB 8800 Ultrotome III microtome. Ultrathin sections were stained with uranyl acetate followed by lead citrate. The samples were visualized using a Zeiss EM 10, a Jeol JEM-1400 or a JEOL 100S.

For negative staining several LSA cells were briefly washed in distilled water and squashed; a drop of the resulting suspension was placed on a Pioloform coated grid. Bacteria were allowed to precipitate for 2–3 min, then a drop of 1% uranyl acetate in distilled water was added for no longer than 1 min. The liquid was then absorbed with filter paper and the grid was air-dried.

For Atomic Force Microscopy the ciliate cells were briefly washed in the sterile lettuce medium, squashed in a small drop of medium on the cover slip and air dried. The images were obtained with a NTEGRA Aura (NT MDT, Russia).

### DNA Extraction and SSU rRNA Genes Characterization

DNA extraction was performed according to Wisotzkey and colleagues [51] for mass cultures (PAR, Pm), or using the protocol for mycelium DNA of NucleoSpin™ Plant DNA Extraction Kit (Macherey-Nagel GmbH and Co., Düren NRW, Germany), for cultures with low cell numbers (In, PS23, LSA, SS03). 18S rRNA gene sequences of host ciliates were then obtained as described elsewhere [52]. The 16S rRNA gene of the endosymbionts were amplified by a PCR reaction employing universal eubacterial primers [53], [54] or by a touchdown PCR reaction [55] employing specifically designed primers: RickFla_F69 5′-GTTAACTTAGGGCTTGCTC-3′, RickFla_F87 5′-CTCTAGGTTAATCAGTAGCAA-3′, RickBas_F166 5′-ATGCTAATGCCGTATATTCTC-3′, Rick_R1270 5′-TTTTAGGGATTTGCTCCACG-3′, Rick_R1455 5′-CCGTGGTTGGCTGCCT-3′. PCR products were then used for library construction or directly sequenced [54].

SSU rRNA gene sequences have been deposited in the European Nucleotide Archive (ENA) with accession numbers HG315605–HG315619.

### Probes Design and FISH Experiments

Preliminary FISH experiments were performed using the eubacterial universal probe EUB338 [56] and the specifically designed probe Rick_697 (5′-TGTTCCTCCTAATATCTAAGAA-3′) in order to verify the presence of endosymbiotic bacteria belonging to the *Rickettsiaceae* family. On the basis of the obtained 16S rRNA gene sequences three probes were designed and synthesized as described elsewhere [57]: TrichoRick_142 (5′-GTTTCCAAATGTTATTCCATAC-3′), targeting all the described symbionts with the exception of that of *Spirostomum minus* SS03; GigaRick_436 (5′-TCATCTTCTCTGCTAAAAGA-3′), targeting only the symbiont of *S. minus* SS03; RickFla_430 (5′-TCTTCCCTGCTAAAAGAACTTT-3′), targeting all the described symbionts. Specificity of the new probes was tested on SILVA [58] and RDP [59] databases. FISH experiments were performed as described by Manz and colleagues [60]; probes were labeled at 5′ with Cy3 or fluoresceine. Slides were then mounted with SlowFade Gold Antifade with DAPI (Invitrogen). The newly designed probes Rick_697, TrichoRick_142, GigaRick_436 and RickFla_430 have been deposited at ProbeBase [61].

### 16S rRNA Gene Sequences Analysis

16S rRNA gene sequences were aligned against those present in the most recent version of the SILVA database [58] using the ARB software package [62].

Phylogenetic analysis was performed on 32 sequences. Sequence lengths were reduced to that of the shortest one, and the long inserts present only in the newly characterized taxa (see Results) were removed (obtaining a 1,338 character matrix); gaps were coded as a fifth character status. Maximum Likelihood (ML) and Bayesian Inference (BI) methods were employed, using the software PHYML [63] and MrBayes [64], respectively. The best substitution model was selected according to the AIC parameter calculated by jModelTest [65]. Bootstrapping (1,000 pseudoreplicates) was applied to the ML analysis. In order to further test the robustness of the results, the same analyses were repeated on modified matrices containing only columns with more than one non-gap character (modified matrix 1; 1,321 characters) or removing all columns containing gaps (modified matrix 2; 1,302 characters). Unless differently stated, similarity values were calculated on the unmodified matrix.

## Results

### Host Identification

The ciliate populations named In and PAR were previously identified as *Euplotes aediculatus* [23] and *Paramecium nephridiatum*, respectively [49]. A partial 18S rRNA gene sequence was obtained for PS23 (1,633 bp), LSA (1,710 bp) and Pm (1,710 bp). In all three cases the sequence showed more than 98% similarity with published sequences of *Paramecium multimicronucleatum* (e. g. strains TH105 and YM25, accession numbers AB252006 and AB252007, respectively [66]) according to NCBI Blastn. Species designation was also confirmed by morphological features like cell size and morphology of the micronuclei as well as the number of the nuclei (data not shown). A partial 18S rRNA gene sequence (831 bp) was also retrieved for population SS03. The highest similarity score (100%) was given in this case by the sequence of *Spirostomum minus* (AM398200; [67]), and the same species designation was suggested by morphological features like size of the cells, oral apparatus position and shape of the macronucleus (data not shown).

### Symbiont Morphology, Ultrastructure and Flagellar Movement

**Figure 1:**
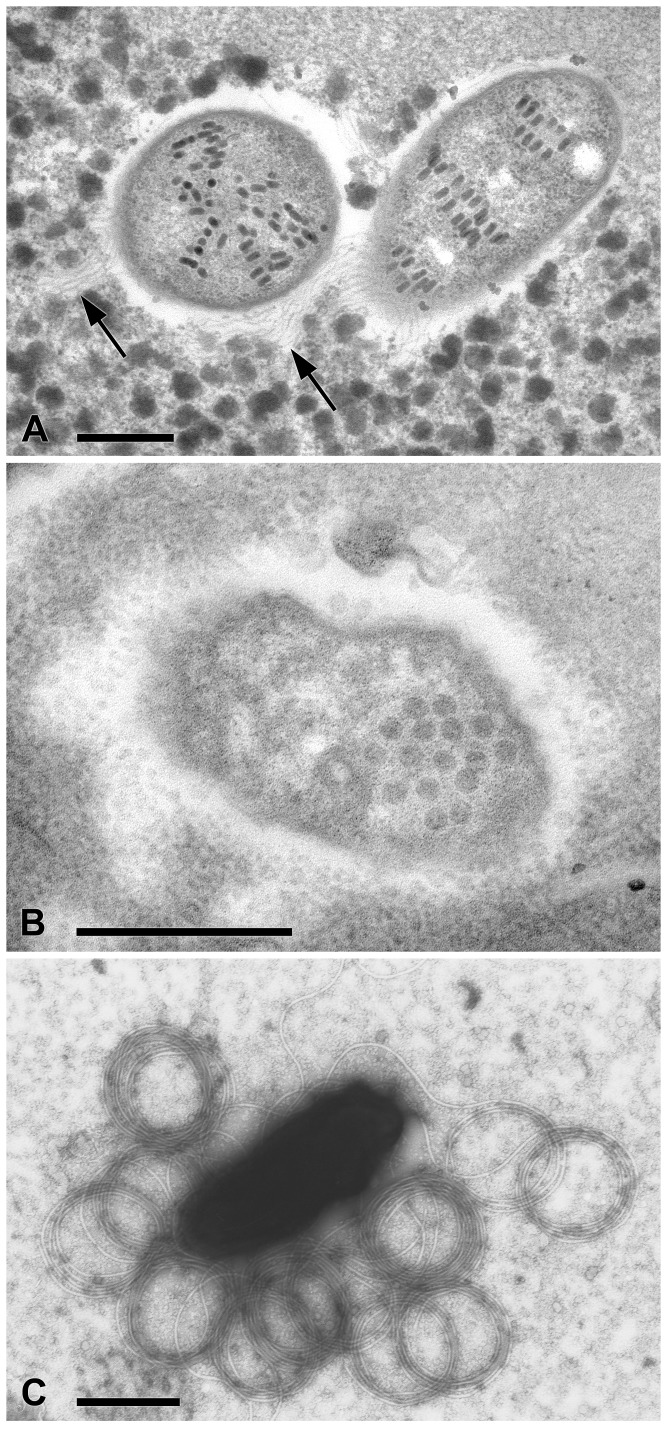
Transmission electron microscopy images of “*Candidatus* Trichorickettsia mobilis”. (A) “*Candidatus* Trichorickettsia mobilis” inside the macronucleus of *P. multimicronucleatum* PS23; several cylindrical electrondense particles, arranged in a regular way, are visible inside the bacterium; arrows indicate the flagella of the symbionts. (B) “*Candidatus* Trichorickettsia mobilis” inside the cytoplasm of *P. nephridiatum* PAR; icosahedral electrondense particles are visible. (C) Negative staining of “*Candidatus* Trichorickettsia mobilis” from *P. multimicronucleatum* LSA: numerous and long flagella are clearly visible. Bars: 0.5 µm.

Morphological and ultrastructural data of all the studied symbionts are summarized in Table 1. Symbionts of *P. multimicronucleatum* (PS23, LSA and Pm) are located almost exclusively inside the macronucleus of the host cell, not surrounded by any symbiosomal vacuole. Most of the bacteria are visible near the inner membrane of the nuclear envelope, while some have been very rarely observed in the cytoplasm of the host ciliate. The short rod-shaped bacterial symbionts possess two membranes arranged in a typical Gram-negative organization (Figure 1A, B). The cytoplasm of the symbionts is slightly electrondense and homogeneous. Highly electrondense, cylindrical corpuscles have been frequently observed inside the bacterial cells. These structures often display a somehow regular arrangement, being aligned in thick bands on the median region of the bacterial cell body. The geometrical shape of these corpuscles, their regular arrangement and their electrondensity suggest that they could be viral capsids (Figure 1A). No other complex structure associated with these capsid-like structures have ever been detected. The presence of numerous peritrichous flagella was detected on sections, on negative contrast and atomic force microscope observations (Figure 1A, C; Figure S1). The flagella are arranged all around the surface of the bacterial cell, they are quite long (more than 10 µ in most cases), often curved and even rolled when fixed (Figure 1C; Figure S1).

*In vivo* observations showed the bacterial symbionts swimming on the edge of the nucleoplasm, displaying a quite fast circular movement (Movie S1), or inside a network of “channels” where the chromatin seems to be less compact or absent (Movies S2, S3). The symbionts were rarely present in the cytoplasm of the host cell and in these cases they have never been observed to swim. On crushed ciliate preparations, the cease of movements has been observed a few minutes after the contact with the external environment. Bacteria trapped inside vesicles originating from the host nuclear envelope did not show any reduction of movement until the dissolution of the vesicle itself.

Symbiotic bacteria from *P. nephridiatum* PAR and of *E. aediculatus* In were instead always present in the ciliate hosts’ cytoplasm and never detected inside either the macro- or the micronucleus. TEM observations performed on *P. nephridiatum* PAR revealed that their morphology resembles that of the *P. multimicronucleatum* symbionts. Viral capsid-like structures were also detected, but their shape and arrangement differ from those of *P. multimicronucleatum* symbionts (Figure 1B). Neither flagella, nor movement have ever been observed in the symbionts of these two ciliate populations.

**Figure 2:**
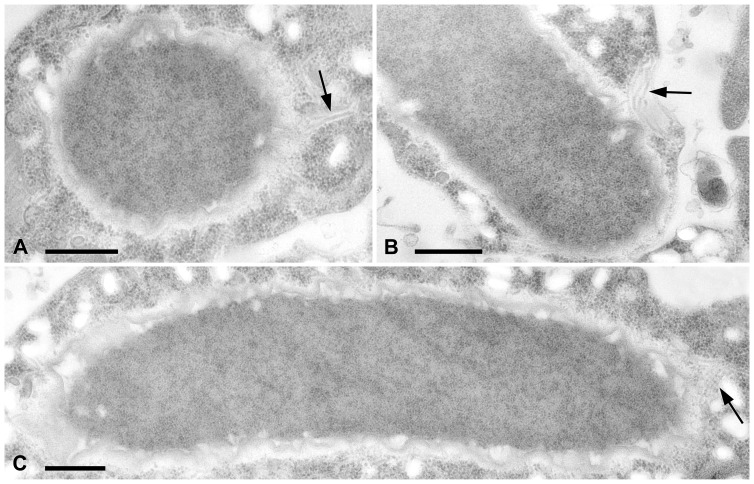
Transmission electron microscopy images of “*Candidatus* Gigarickettsia flagellata” in transverse (A) and longitudinal (B, C) sections. Arrows indicate the flagella. Bars: 0.5 µm.

Bacterial symbionts of *S. minus* SS03 are also located in the cytoplasm of the host cell. Their most striking feature is the remarkable cell size, with some specimens attaining 20 µm in length and 1.2 µm in width (Figure 2). Their cytoplasm is generally highly electrondense and homogeneous, the membrane organization resembles that of typical Gram-negative bacteria. No viral capsid-like structures were observed inside the bacterial cells, but the presence of flagella was shown by TEM observations (Figure 2). Despite their huge size, these symbionts constantly swim in the cytoplasm of the host cell. Their movements are not erratic, but straightforward and parallel to the host cell membrane. Their cell body is flexible and bends to continue the straight movement when they reach the host cell extremities. Reversion of the direction of movement has never been observed. Rather often host organelles (e. g. nuclei) are touched or even misplaced by bacteria during their rushes. Despite this, host ciliates do not show any obvious sign of stress. Bacterial movement ceases almost immediately after contact with the external environment (e. g. after host cell disruption).

### 16S rRNA Gene Characterization of Symbionts and FISH Experiments

Almost full-length 16S rRNA gene sequences of the symbionts were obtained from all the examined samples, with a length ranging from 1,314 bp to 1,624 bp (accession numbers HG315609–HG315619). The most peculiar feature of the obtained sequences is the presence of two quite long inserted elements in positions 207 and 212 (*Escherichia coli*), respectively. They are separated only by a 4 bp long region (consensus sequence: 5′-TTTA-3′) present in all other sequences included in the analysis. These insertions are not present in any other 16S rRNA gene sequence obtained from bacteria belonging to the order *Rickettsiales.*

Sequences obtained from *Paramecium* (PAR, PS23, Pm, LSA) and *Euplotes* (In) samples show an insertion of 48 bp in position 207 and another one of 152 bp in position 212. The concatenated sequences of the two insertions share a similarity value range of 90.7–100% among different strains. This range is quite larger than that calculated among the same strains using the entire sequence (98.4–100%), or the sequence without the insertions (99.7–100%). A Blastn search using the concatenated sequence of the insertions provided only low-score hits (72–88% similarity, and 9e^−8^-2e^−10^ e-values for the best 10 hits) from genomic sequences of *Rickettsia* species. The most represented homolog is the gene for the beta subunit of RNA polymerase. All these data suggest that the insertions were transferred as mobile elements from other genomic regions of a *Rickettsia*-like organism (likely the symbiont itself). Although further investigations are required in order to verify this point, they could be non-functional and subjected to weak purifying selection.

The consensus sequence of clones obtained from the symbiont of *S. minus* SS03 possesses two shorter insertions of 23 bp each.

Once the insertions are removed, the similarity between the sequence of SS03 symbiont and the sequences of other symbionts here described is 95.0–95.1%. Similarities shared with other members of *Rickettsiaceae* are below 94.0% (SS03 symbiont) and below 97.32% (other symbionts).

The probe Rick_697 matched 249 16S rRNA gene sequences on SILVA database. 90% of these sequences belongs to the *Rickettsiaceae* family, while the remaining 10% includes *Wolbachia pipientis*, the bacterial symbiont of *Sitophilus zeamais* and some uncultured bacteria. The same probe matched 626 sequences on RDP database, 95% of which belonging to members of the *Rickettsiaceae* family and the rest to uncultured bacteria. Probes specifically designed for the newly characterized symbionts were shown to be highly specific. Indeed, probe RickFla_430 matched no sequence on SILVA database and only 8 sequences on RDP (all belonging to unclassified organisms), probe TrichoRick_142 matched no sequence on SILVA and only 3 sequences on RDP (all from unclassified organisms) and probe GigaRick_436 matched no sequences on SILVA nor on RDP.

**Figure 3:**
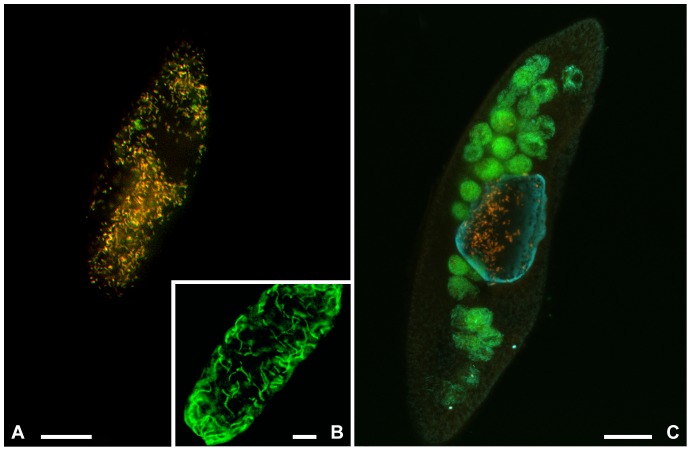
FISH experiments performed on “*Candidatus* Trichorickettsia mobilis” and “*Candidatus* Gigarickettsia flagellata”. Symbionts inside *P. nephridiatum* PAR (A) and inside *P. multimicronucleatum* LSA (C) with probe RickFla_430 (Cy3, red signal) together with eubacterial probe EUB338 (fluoresceine, green signal). Food bacteria, labeled only in green, are visible inside food vacuoles in LSA (C). FISH experiment performed on “*Candidatus* Gigarickettsia flagellata” with probe GigaRick_436 (green signal) is shown in B. Bars: 20 µm.

In order to confirm 16S rRNA gene characterization of the newly described symbionts, FISH experiments were performed using the specifically designed probes (Figure 3, Figure S2). While probe RickFla_430 gave a bright and sharp signal inside ciliate cells of all the studied samples, probe TrichoRick_142 gave a positive signal only on samples of *P. multimicronucleatum* PS23, Pm and LSA, *P. nephridiatum* PAR and *E. aediculatus* In. On the contrary, probe GigaRick_436 gave a positive signal only inside the cytoplasm of *S. minus* SS03.

### Phylogeny of Symbionts

All the 16S rRNA gene sequences obtained in this work are tightly associated in a highly supported cluster, with the exception of the sequence from the *S. minus* SS03 symbiont. This bacterium does form a monophyletic group with the others here characterized, but stands at the top of a much longer branch. Moreover, statistical support to the group including all new sequences is relatively low (70/0.98 in the analysis performed on the non-modified matrix; up to 75/0.99 with the modified matrix 2).

**Figure 4:**
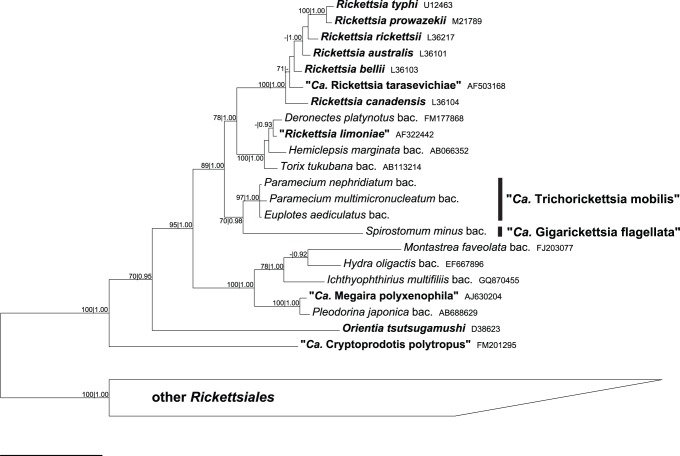
Bayesian Inference tree of the family *Rickettsiaceae*. The tree was built on the unmodified character matrix (see text) employing the GTR+I+G (8 gamma categories) model. Numbers associated to nodes represent Maximum Likelihood bootstraps and Posterior Probabilities, respectively (values below 70|0.85 are omitted). Taxa that received a formal or provisional binomial name are in bold. The bar stands for an inferred sequence divergence of 5%. “*Ca.*”, “*Candidatus*”; “bac.”, “bacterium associated to”.

The topology of the ingroup does not differ between ML and BI analyses, and shows only minor differences in unsupported nodes when calculated on modified matrices. The clade formed by the ciliate endosymbionts characterized herein is the sister group of validly described *Rickettsia* species, exclusively found in arthropods, and related organisms that were not formally described (e. g. “*Rickettsia limoniae*”) and that possess a wider host spectrum. Other bacterial symbionts of ciliates belonging to the *Rickettsiaceae* family are more distantly related, and scattered in different clades together with symbionts of other aquatic organisms (Figure 4).

## Discussion

### Features and Characterization of the Novel Symbionts

According to the obtained data, the characterized bacterial symbionts unambiguously belong to the order *Rickettsiales* and to the family *Rickettsiaceae*. The same data, together with similarity analysis, indicate that we are dealing with two distinct bacterial species [68]. The first one, represented by the symbionts detected in samples of *Paramecium* (PS23, Pm, LSA, PAR) and *Euplotes* (In), will be thereafter referred to as “*Candidatus* Trichorickettsia mobilis”. The second one is represented by the symbiont of *S. minus* (SS03) and will be thereafter referred to as “*Candidatus* Gigarickettsia flagellata”, distinctive for its much larger size, different ultrastructural features and shorter insertion elements in the 16S rRNA genes.

Obtained results show that “*Candidatus* Trichorickettsia mobilis” inhabits the macronucleus and possesses flagella and motility inside *P. multimicronucleatum.* It is worthy of mention that a flagellated endosymbiont showing the same morphological features and moving ability inside the host macronucleus was briefly described in the past without any molecular characterization in *P. multimicronucleatum* populations isolated in Boston, USA, and Moldova [69]. On the other hand, “*Candidatus* Trichorickettsia mobilis” is localized in the cytoplasm and does not show either flagella or motility inside *P. nephridiatum* and *E. aediculatus*. Viral capsid-like structures were found in several samples, but according to morphology they belong to at least two different types. The characterization of this bacterium from several different samples pointed out the possible expression of remarkably different features within the same bacterial species. Differences in intracellular localization and moving ability indicate a certain degree of plasticity, apparently driven by the host species.

Nuclear localization has already been reported for bacteria belonging to the family *Rickettsiaceae*. Indeed, members of the Spotted Fever Group (SFG) in the genus *Rickettsia* were already known to reside also in the nucleus of the eukaryotic host cell [70]. Moreover, Ogata and colleagues [40] reported for the species *Rickettsia bellii* the capability of colonizing the host cell nucleus and dividing inside it. It has been speculated that the nucleus can represent both a protected, nutrient-rich environment to be exploited for these endosymbiotic bacteria and an optimal location for possible interference with host gene expression [71]. Other members of the orders *Rickettsiales* that typically invade the host nuclei are the ciliate symbionts belonging to the family *Holosporaceae* [22], [72] and the genus *Caedibacter* [19], [73].

### Flagellar Motility and its Implications

The most surprising feature of the two newly described bacterial species is the unambiguous presence of active flagellar movement. This is an absolute novelty not only in the family *Rickettsiaceae*, but also in the entire order *Rickettsiales*. Available data indicate that flagellar movement could represent an ancestral character for all the *Alphaproteobacteria*, which was secondarily lost in *Rickettsiales* [74]. The finding of flagella and of vertically inherited flagellar genes in “*Candidatus* Midichloriaceae” family [46], [48] and, now, the finding of flagellar movement in two bacteria belonging to the family *Rickettsiaceae* suggest that not only the genes, but also their full expression and functioning, have been retained with a spotted pattern in the different evolutionary lineages of the order. According to this scenario, the possibility that the ancestor of *Rickettsiales* displayed flagellar movement re-evaluates the hypothesis that motility played a key-role in the origin of mitochondria.

Alternatively, it could be also hypothesized that flagellar movement has been re-acquired by the symbionts here described. In this case, a horizontal gene transfer could have occurred, for example, with some other bacterium associated to protist ciliates. Indeed, horizontal gene transfer between bacteria associated to protists has already been documented [40]. The presence of electrondense particles, resembling viral capsids, which has been shown inside “*Candidatus* Trichorickettsia mobilis”, suggests also the possibility of gene exchange involving a viral partner, although the presence of flagella and capsid-like structures do not always correlate (Table 1). The acquisition of additional information on these particles and on their possible activity will help address this issue. To summarize, an independent re-acquisition of flagella by different members of the order cannot be excluded, but its explanation requires at present several speculative assumptions. Therefore, all these hypotheses need to be tested by genome characterization and analysis, an approach that has only recently been applied to bacterial symbionts of ciliates [75]. Moreover, further screenings on non-conventional (i. e. non-blood-feeding arthropods) potential hosts from different environments could enlarge the record of motile *Rickettsia*-related bacteria. This would possibly shed light on the evolutionary path of this peculiar feature and its adaptive meaning. In any case, data here reported definitively invalidate the hypothesis of a complete absence of such an important character in the entire order.

### Diversity of *Rickettsiales* Bacteria in Ciliates

Symbiotic systems involving protists as hosts are turning out to be a precious source of information on the diversity and evolution of bacteria belonging to the order *Rickettsiales*. Our results strongly suggest the hypothesis that several ancestral features of *Rickettsiales* could have been retained mainly in bacteria colonizing unicellular hosts [33]. In the light of the new findings here reported, the hypothesis that protists could represent the ancestral hosts of the *Rickettsiales* should be perhaps re-evaluated and better investigated.

Beside evolutionary aspects, some ecological considerations merit attention. The two new bacterial species here described have been detected in samples derived from aquatic habitats. Considering that all the best-known *Rickettsiaceae* bacteria were isolated from terrestrial habitats, this means that the new species inhabit a completely different environment. This finding has to be added to some recent reports of other bacteria belonging to the family both from marine [12], [15] and freshwater habitats (see for example [3], [4], [11], [13]). Taking all these data into account, it seems evident that the aquatic environment represents a well-exploited and important habitat for bacteria of the family *Rickettsiaceae*. Moreover, it is noteworthy that two of the studied population (PAR and Pm) were sampled in waste water treatment plants. Perhaps, protists colonizing such highly impacted environments should deserve more attention as possible reservoirs of bacteria related to pathogenic groups.

### Taxonomy and Diagnosis

According to the guidelines of Fournier and Raoult [76] a bacterial species can be included in the genus *Rickettsia* only if its 16S rRNA gene sequence shows a similarity value of 98.1% or higher with respect to other members of the genus. Following this rule, some new bacteria of the family *Rickettsiaceae*, like the yet-undescribed “*Rickettsia limoniae*” and others which have been recently characterized (see for example [10], [11]), should not be considered representatives of the genus *Rickettsia*. For this reason, we describe the two newly characterized bacteria as two novel bacterial genera within the family *Rickettsiaceae*. Their classification in two different genera is a precaution due to the conspicuous sequence differences between them, and the relatively low statistical support underlying their association in phylogenetic analysis. The putative clade formed by these taxa can be identified through the FISH oligonucleotide probe RickFla_430 (5′-TCTTCCCTGCTAAAAGAACTTT-3′) and the presence of insertion elements in the 16S rRNA gene, although its monophyly should be further tested.

Description of “*Candidatus* Trichorickettsia mobilis”. *Trichorickettsia mobilis* (Tric.ho.ric.ket’tsi.a mo’bi.lis; Gr. masc. n. *thrix*, hair, N.L. fem. n. *Rickettsia*, from the name of a related genus, N.L. fem. n. *Trichorickettsia*, hairy *Rickettsia*; L. adj. *mobilis*, motile). Rod-shaped bacterium, up to 2.6 µm in length and 1.3 µm in width. Macronuclear or cytoplasmic symbiont of protist ciliates of the genera *Paramecium* and *Euplotes*. Displaying flagella and swimming behavior inside the host cell of *P. multimicronucleatum*. Electrondense cytoplasm, frequently hosting particles resembling viral capsids with various shapes arranged in regular fashions. Belonging to the family *Rickettsiaceae* in the order *Rickettsiales*. Basis of assignment: 16S rRNA gene sequence (accession number: HG315612) and positive match with the specific FISH oligonucleotide probe TrichoRick_142 (5′ – GTTTCCAAATGTTATTCCATAC -3′). Uncultured thus far.

Description of “*Candidatus* Gigarickettsia flagellata”. *Gigarickettsia flagellata* (Gi.ga.ric.ket’tsi.a fla.gel.la’ta; Gr. masc. n. *gigas*, giant, N.L. fem. n. *Rickettsia*, from the name of a related genus, N.L. fem. n. *Gigarickettsia*, giant *Rickettsia*; L. adj. *flagellata*, with flagella). Long rod shaped bacterium, up to 20 µm in length and 1.2 µm in width. Cytoplasmic bacterial symbiont of *Spirostomum minus* (Ciliophora, Heterotrichea). Possessing flagella and swimming behavior inside the host cell. Belonging to the family *Rickettsiaceae* in the order *Rickettsiales*. Basis of assignment: 16S rRNA gene sequence (accession number: HG315613) and positive match with the specific FISH oligonucleotide probe GigaRick_436 (5′-TCATCTTCTCTGCTAAAAGA-3′). Uncultured thus far.

## Supporting Information

Figure S1 **Atomic force microscope image of “** ***Candidatus*** **Trichorickettsia mobilis” from** ***P. multimicronucleatum*** **LSA.** Flagella are clearly visible.
(JPG)

Figure S2 FISH performed on “*Candidatus* Trichorickettsia mobilis” inside the macronuclei of *P. multimicronucleatum* Pm (A) and *P. multimicronucleatum* PS23 (C), and the cytoplasm of *E. aediculatus* In (B) with the probe RickFla_430 (red signal) and, only in (C), with the eubacterial probe EUB338 (green signal). Bars: 10 micrometers.
(JPG)

Movie S1 **“** ***Candidatus*** **Trichorickettsia mobilis” swimming on the edge of the nucleoplasm of** ***P. multimicronucleatum*** **PS23.**
(MP4)

Movie S2 **“** ***Candidatus*** **Trichorickettsia mobilis” swimming inside the macronucleus of a crushing ciliate cell of** ***P. multimicronucleatum*** **PS23.**
(MP4)

Movie S3 **“** ***Candidatus*** **Trichorickettsia mobilis” swimming inside the macronucleus of** ***P. multimicronucleatum*** **LSA.** The beating of somatic and oral ciliature of the protist host is also visible.
(MP4)
