# Supplementary figures and images for: Flagellar Movement in Two Bacteria of the Family Rickettsiaceae: A Re-Evaluation of Motility in an Evolutionary Perspective
Source: PLoS One. 2014 Feb 5;9(2):e87718. doi: 10.1371/journal.pone.0087718 (PMC3914857; doi:10.1371/journal.pone.0087718)

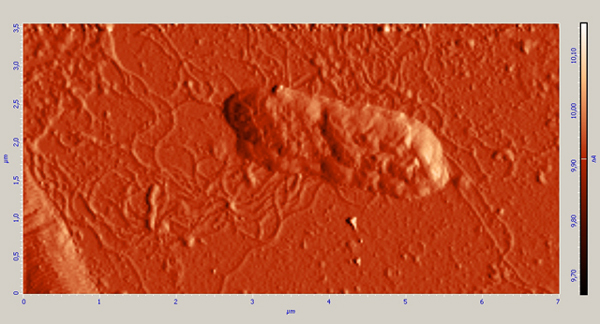

Supplement: Figure S1 — Atomic force microscope image of “ Candidatus Trichorickettsia mobilis” from P. multimicronucleatum LSA. Flagella are clearly visible. (JPG) [file pone.0087718.s001.jpg]

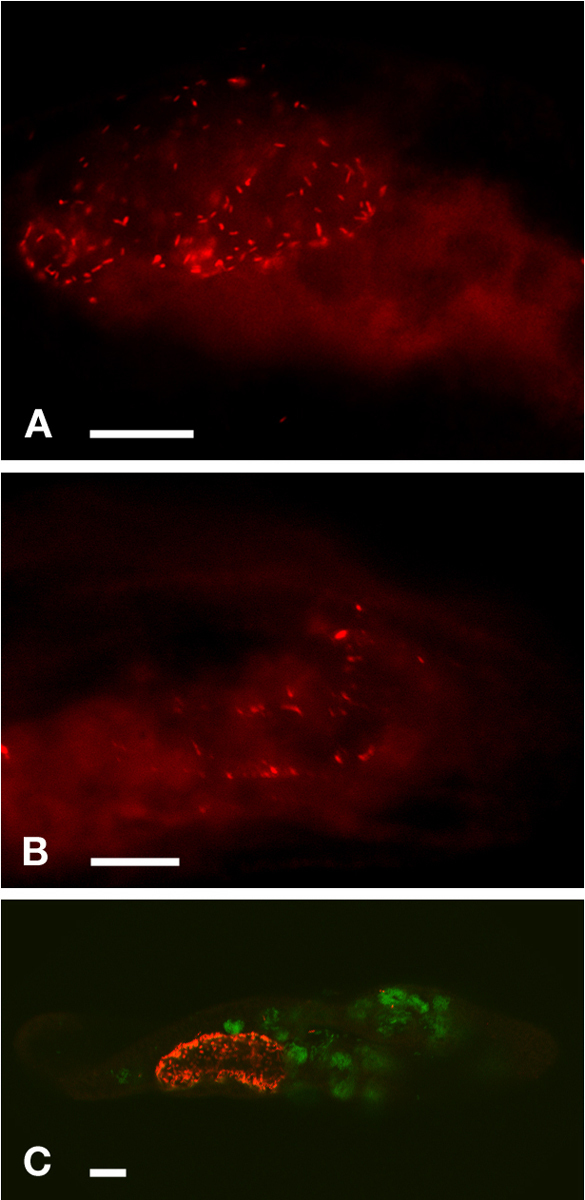

Supplement: Figure S2 — FISH performed on “Candidatus Trichorickettsia mobilis” inside the macronuclei of P. multimicronucleatum Pm (A) and P. multimicronucleatum PS23 (C), and the cytoplasm of E. aediculatus In (B) with the probe RickFla_430 (red signal) and, only in (C), with the eubacterial probe EUB338 (green signal). Bars: 10 micrometers. (JPG) [file pone.0087718.s002.jpg]
